# Increasing community capacity to prevent childhood obesity: challenges, lessons learned and results from the Romp & Chomp intervention

**DOI:** 10.1186/1471-2458-10-522

**Published:** 2010-08-31

**Authors:** Florentine P de Groot, Narelle M Robertson, Boyd A Swinburn, Andrea M de Silva-Sanigorski

**Affiliations:** 1 WHO Collaborating Centre for Obesity Prevention, Deakin University, 1 Gheringhap Street, Geelong, Victoria 3220, Australia; 2 Jack Brockhoff Child Health & Wellbeing Program, Melbourne School of Population Health, The University of Melbourne, Australia

## Abstract

**Background:**

Obesity is a major public health issue; however, only limited evidence is available about effective ways to prevent obesity, particularly in early childhood. *Romp & Chomp *was a community-wide obesity prevention intervention conducted in Geelong Australia with a target group of 12,000 children aged 0-5 years. The intervention had an environmental and capacity building focus and we have recently demonstrated that the prevalence of overweight/obesity was lower in intervention children, post-intervention. Capacity building is defined as the development of knowledge, skills, commitment, structures, systems and leadership to enable effective health promotion and the aim of this study was to determine if the capacity of the Geelong community, represented by key stakeholder organisations, to support healthy eating and physical activity for young children was increased after *Romp & Chomp*.

**Methods:**

A mixed methods evaluation with three data sources was utilised. 1) Document analysis comprised assessment of the documented formative and intervention activities against a capacity building framework (five domains: Partnerships, Leadership, Resource Allocation, Workforce Development, and Organisational Development); 2) Thematic analysis of key informant interviews (n = 16); and 3) the quantitative Community Capacity Index Survey.

**Results:**

Document analysis showed that the majority of the capacity building activities addressed the Partnerships, Resource Allocation and Organisational Development domains of capacity building, with a lack of activity in the Leadership and Workforce Development domains. The thematic analysis revealed the establishment of sustainable partnerships, use of specialist advice, and integration of activities into ongoing formal training for early childhood workers. Complex issues also emerged from the key informant interviews regarding the challenges of limited funding, high staff turnover, changing governance structures, lack of high level leadership and unclear communication strategies. The Community Capacity Index provided further evidence that the project implementation network achieved a moderate level of capacity.

**Conclusions:**

*Romp & Chomp *increased the capacity of organisations, settings and services in the Geelong community to support healthy eating and physical activity for young children. Despite this success there are important learnings from this mixed methods evaluation that should inform current and future community-based public health and health promotion initiatives.

**Trial Registration Number:**

ANZCTRN12607000374460

## Background

Health promoters and public health practitioners have existing knowledge about what 'works' in health promotion, however the evidence suggests that the majority of the positive outcomes are not sustained [1]. The focus therefore needs to be on ways to make the impacts of public health and health promotion initiatives sustainable, and community capacity building has emerged as one such approach [2]. Community capacity building is about developing sustainable skills, organisational structures, resources and commitment to health improvement in health and other sectors, and prolonging and multiplying health gains many times over [3]. Several resources have been developed for the integration of community capacity building into health promotion practice through project implementation (or 'action') plans (e.g. the New South Wales Health Capacity Building framework) [4] and this approach is now gaining ground in public health nutrition and obesity prevention [5,6].

In the context of increasing childhood obesity [7-9] and lack of effective intervention strategies for young children, the obesity prevention demonstration project, *Romp & Chomp *was established. This project was a community-wide obesity intervention implemented from 2005-2008 with wide stakeholder involvement and funding from the Victorian state government (AU$ 111,200). The target group was 12,000 children aged 0-5 years in the regional City of Greater Geelong (CoGG) in Victoria, Australia. *Romp & Chomp *had a multi-strategy, multi-settings, community capacity building approach and the intervention program was designed, planned and implemented by several key stakeholder organisations, particularly Barwon Health, CoGG, Geelong Kindergarten Association, Leisure Networks, the Department of Human Services (DHS), Deakin University, Bellarine Community Health, Dental Health Services Victoria, and Kids-'Go for your life'. Deakin University also provided support, training and evaluation of the project. A management committee of stakeholders oversaw the implementation of the action plan and assisted the project coordinators (employed through Barwon Health and DHS) to fulfil their duties.

*Romp & Chomp *intervention strategies were developed with a strong focus on developing sustainable changes in areas of policy, socio-cultural, physical and economic environments. The action plan had an overarching aim (To increase the capacity of the Geelong community to promote healthy eating and active play and to achieve healthy weight in under 5s') and eight project objectives, with a number of strategies detailed to achieve the objectives. Alongside the intervention was a comprehensive and multi-level evaluation [10] and we have recently demonstrated that post-intervention, the prevalence of overweight and obesity was significantly lower in intervention children (prevalence declined by 2.5% and 3.4% for 2 and 3.5 year old children respectively) compared to a comparison sample of children (prevalence declined by 0.7% in both age groups) [11]. In addition, positive differences in the diets of children from the intervention sample were also found [11].

The focus of this paper is the evaluation of *Romp & Chomp *objective 1: 'to increase the capacity of relevant Geelong organizations to promote healthy eating and active play'. In *Romp & Chomp *the capacity building strategies were related to increasing leadership, training and funding into the community as catalysts for a cyclic and expanding process of community and organisational change [10]. This paper aims to determine if the capacity of the Geelong community, represented by key stakeholder organisations, to support healthy eating and physical activity for young children was increased after *Romp & Chomp*.

## Methods

### Data Triangulation

Triangulation (multiple methods of data collection) was used to increase confidence in the research findings [12]. This involved integrating the results from three mixed methods (document analysis, interviews with key informants, and the Community Capacity Index) to determine the degree of consistency in the results. Inconsistency was not seen to weaken credibility, but rather viewed as informative and illuminative [13,14].

### Document analysis

All available *Romp & Chomp *documentation was examined for this study. The documents included steering and management committee minutes, all eight versions of the project implementation (action) plans, grant applications, protocols, formative process evaluation, focus group evaluations and project reports. The information from these documents also guided the development of the questions for the semi-structured key informant interviews.

#### Action Plan Assessment

The *Romp & Chomp *action plans listed the strategies and associated actions for the implementation of the intervention. These were developed based on stakeholder consultation [15], evidence available in the literature, previous experiences, knowledge within the project group and consultation with early childhood workers across the four participating early childhood services (Preschool, Family Day Care, Long Day Care and Maternal & Child Health). The action plan was revised throughout the intervention implementation period in accordance with staff turnover, level of capacity, resources available, and the perceived needs of the project management committees. For this study, all versions of the action plans were assessed against the New South Wales (NSW) Capacity Building Framework [4]. This framework was developed to guide effective capacity building practice within health promotion and contains five domains (Partnerships, Leadership, Resource Allocation, Workforce Development and Organisational Development) [4]. In our study this framework was used as a tool to evaluate the intervention strategies designed to build community capacity by mapping the actions documented in the *Romp & Chomp *action plan into the five domains of this framework. The process was performed by two researchers independently, whereupon outcomes were compared and consensus reached.

### Key informant interviews

Key informant interviews (n = 16) were conducted at the end of the *Romp & Chomp *intervention to gain further insights into the intervention implementation and the ongoing ability of the network implementing and sustaining this project to support healthy eating and physical activity for children. Key informants were identified by the evaluation manager and included individuals from each of the partner organisations who had worked closely with *Romp & Chomp *or had a significant influence on the project. The key informants were invited to participate in a semi-structured interview and to complete the Community Capacity Index survey (details below). Interview questions concerned the interviewee's role and experiences within the intervention project, communication, sustainability of partnerships and advice for future similar projects. Example questions are: "*What was your role within the Romp & Chomp project?"; "What were the major achievements that were made?"; *and *"Can you describe the communication that existed between the steering and management committees?"*. Of the seventeen stakeholders approached sixteen were accessible (94%) and interviewed in-person by two researchers, one acting as the primary interviewer and the other was there to act as the primary note taker and to ensure consistency across interviews. The roles were alternated for each interview and the interviews were transcribed and verified. Thematic analysis of the transcripts was conducted [16] and verified by a second researcher.

### Community Capacity Index

The Community Capacity Index (CCI) was used to provide a quantitative assessment of the implementation network at the end of the project. It is difficult to find an instrument that fully captures changes in community capacity and can be used in program evaluation. We chose to use the CCI as one method in a mixed method evaluation of changes in community capacity, alongside the methods described above. The CCI is an evidence-based tool, developed by the University of Queensland and has been trialled in several projects and validated for use in this context [17]. The index examines community capacity across four domains: Network Partnerships (the relationships between the organisations within the community network); Knowledge Transfer (the development, exchange and use of information within and between the organisations and groups within the community network); Problem Solving (the ability to identify and solve problems arising in the development and implementation of the program); and Infrastructure (the level of investment in the network by the organisations). For each of the first three domains there are three levels of capacity, for the remaining domain (infrastructure) there are four levels of capacity. Undertaking particular activities and the presence of specific abilities (as reported by participants) provides an indication of the level of capacity achieved. These were captured by individual and aggregated indicators. The response categories available for participants to rate the network's capacity to achieve a range of criteria were: not at all/very limited; somewhat; substantial; and almost entirely/entirely. For the first three domains, the interpretation of the results are that the higher the achieved capacity, the greater the sustainability of the network. The fourth domain (Infrastructure) has four sub-domains: Policy Investments, Financial Investments, Human/Intellectual Investments and Social Investments, and also indicates sustainability. All key informants were asked to complete the self-administered CCI questionnaire prior to their interview and a response rate of 50% was achieved.

## Results

### Action Plan assessment

Table 1 shows that close to 40% of all intervention activities (as detailed in the *Romp & Chomp *action plans) were related to building partnerships, with all the strategies targeted to the first two elements of the Partnership domain (shared goals and relationships). Multiple, appropriate partners were identified and relationships were established through documented agreements and continued maintenance of relations with other projects and organisations. There were no documented strategies addressing the planning, implementation, evaluation or sustainability elements of the partnership domain. There were also no documented strategies to enhance the building of leadership within *Romp & Chomp*. A significant proportion of the actions in the *Romp & Chomp *action plan aggregated under the Resource Allocation domain, although within this domain, there were no actions related to the decision-making tools and physical resources elements. Only a small number of project activities were related to the Workforce development domain and these were not evenly distributed across the elements. None of the documented intervention activities were mapped into the Leadership domain.

**Table 1:** *Romp & Chomp *intervention activities mapped into the New South Wales capacity building framework

Framework domains and elements	Intervention activities; n (%)
**Partnerships**	**21/53 (39.6%)**
*Shared goals*	*6/21 (28.6%)*
*Relationships*	*15/21 (71.4%)*
*Planning*	*0/21 (0%)*
*Implementation*	*0/21 (0%)*
*Evaluation*	*0/21 (0%)*
*Sustained outcomes*	*0/21 (0%)*
**Leadership**	**0/53 (0%)**
*Interpersonal skills*	*0/0 (0%)*
*Technical skills*	*0/0 (0%)*
*Personal qualities*	*0/0 (0%)*
*Strategic visioning*	*0/0 (0%)*
*Systems thinking*	*0/0 (0%)*
*Visioning the future*	*0/0 (0%)*
*Organisational management*	*0/0 (0%)*
**Resource Allocation**	**12/53 (22.6)**
*Financial resources*	*3/12 (25%)*
*Human resources*	*1/12 (8.3%)*
*Access to information*	*3/12 (25%)*
*Specialist advice*	*2/12 (16.7%)*
*Decision making tools and models*	*0/12 (0%)*
*Administrative support*	*3/12 (25%)*
*Physical resources*	*0/12 (0%)*
**Workforce development**	**4/53 (7.5%)**
*Workforce learning*	*1/4 (25%)*
*External courses*	*1/4 (25%)*
*Professional development opportunities*	*2/4 (50%)*
*Education/Under- and Postgrad degrees*	*0/4 (0%)*
**Organisational Development**	**16/53 (30.2)**
*Policies and procedures*	*1/16 (6.3%)*
*Strategic directions*	*0/16 (0%)*
*Organisational structures*	*5/16 (31.2%)*
*Management support*	*6/16 (37.5%)*
*Recognition and reward system*	*0/16 (0%)*
*Information systems*	*4/16 (25%)*
*Quality Improvement systems*	*0/16 (0%)*
*Informal culture*	*0/16 (0%)*
	**Total 53 (100%)**

In total there were 53 actions in the action plan. ^a^Score per domain is the proportion of actions in the action plan per NSW Framework domain. ^b^Score per element is the proportion of actions in the action plan per NSW Framework element.

### Key Informant Interview themes

Three major themes emerged from the key informant interviews. These were related to relationships, resources and project structures. Each theme is discussed below.

**Relationships -** The interviewees described the major positive outcomes from the project as the great achievement of bringing together the big 'players' from across the Geelong community to work together, the establishment of partnerships with other similar projects, and the sustainable relationships that arose:

*"...we looked at bringing this all together in a more strategic approach and that became the basis for developing the Children's Health and Wellbeing strategy group"*.

There were also a number of negative reactions that may reflect the lack of processes and protocols that could have facilitated better partnerships and overcome philosophical differences between partners about the project:

*"...we're dealing with large organizations and inevitably multiple personalities with different ideas about how things should be done"*.

There were also negative feelings about the perception that some partner organisations tried to hold onto the ownership and branding of their own projects. A frequently mentioned problem during the interviews was a lack of project leadership which may be a consequence of high staff turnover as throughout the intervention period a total of five project coordinators were employed.

**Resources -** There was a strong feeling of frustration related to the lack of resources and funding available for project implementation, although several key formants identified this as an advantage and closer to the real world situation:

"..trying it on a shoestring budget.."

A number of strategies were utilised to increase capacity and sustainability throughout the project. The incorporation of Active Play in the TAFE (Technical and Further Education) curriculum was, viewed as one of the positive and sustainable outcomes from the project, as were the development and adoption of healthy eating and physical activity policies in early childhood settings. The intervention strategy of training allied health professionals (dieticians, physiotherapists, dental staff, occupational therapists etc) to support the health promotion activities in the kindergartens was also viewed positively, although the sustainability of this part of the intervention was questioned. The lack of skills and knowledge of some of the committee members related to capacity building and health promotion was also mentioned during the interviews, but the involvement of experts was rated positively.

**Structures -** Key informants made a number of comments regarding the lack of organisational structures and management support throughout the life of the project. There was seen to be ambiguity about the roles and responsibilities of individuals, organisations and the various committees. Comments were also made about the lack of meetings of the higher level reference group and the fact that the project managers and steering committee felt kept on a tight rein and unable to make independent decisions, which was seen to have slowed down processes:

*"When you've got people that are managers and are quite capable of making those decisions....are we supposed to be just steering the project and not making decisions?"*.

### Community Capacity Index (CCI)

**Figure 1:**
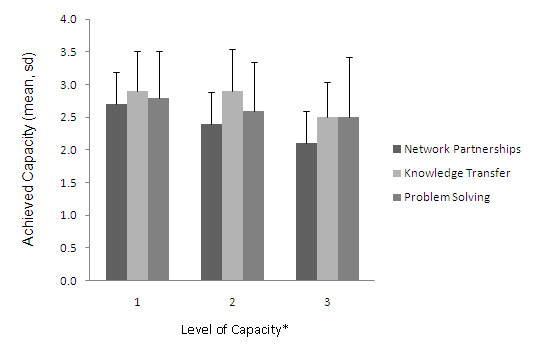
**Mean achieved Capacity in the 3 levels of Network Partnerships, Knowledge Transfer and Problem Solving**. Network Partnership; level 1: identify partners, level 2: deliver program, level 3: maintain network. Knowledge Transfer; level 1: develop program, level 2: transfer, level 3: integrate in mainstream practice. Problem Solving; level 1: working together, level 2: identify and overcome problems, level 3: sustain.

**Figure 2:**
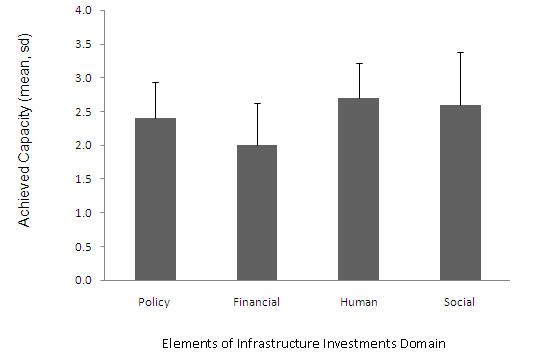
**Mean achieved capacity of the four types of Infrastructure Investments**.

In the CCI there are three levels of capacity for each domain, with the levels related to increasing sustainability of the specific domain activities. A score of 3 represents substantial capacity and a score of 4 represents capacity entirely reached [18]. Figure 1 shows that in the Network Partnerships domain, there is a higher mean score in the first level (related to identification of members of the network), a lower score (and therefore less capacity) to deliver the project (level 2) and even less to maintain and resource the project (level 3). The scores across the levels are more even within the Knowledge Transfer domain, which also has the highest scores compared to the other domains; indicating the network had substantial capacity to develop and implement the project (level 1 and 2) but a lower capacity to integrate the project into mainstream practices. The outcomes for the Problem Solving domain show a similar pattern; with a score close to 'substantial' capacity for the network to work together to solve problems and to identify and overcome problems (level 1 and 2), and a lower capacity to sustain flexible problem solving (level 3). Overall, the scores were below three, which suggests that the achieved capacity has not reached the 'substantial' level. In figure 2 we see the rating of the Infrastructure Investments domain. The financial investments element scored very low indicating that there was only limited capacity to develop financial capital. The capacity to develop policy and social capital scored higher, and the Human element scored the highest in this domain, indicating the network had a high capacity to develop human/intellectual capital.

## Discussion

*Romp & Chomp *was a large-scale, community-wide intervention project that aimed to increase the capacity of the Geelong community to promote healthy eating and physical activity for young children. The results from this mixed methods study demonstrate that this was achieved in only some of the domains of capacity building, specifically those related to partnerships, organisational development and resource allocation. However significant areas of capacity building were not addressed during the project implementation, particularly related to high-level and ongoing leadership. There were a number of positive outcomes identified in this study related to capacity building, including the establishment of sustainable partnerships, use of specialist advice, and integration of activities into ongoing formal training for early childhood workers. A number of challenges were also identified which related predominately to budgetary constraints, staff turnover, unclear governance structures, lack of ongoing high level leadership and inadequate communication between the partnering organisations. Despite these challenges however, the capacity of the Geelong community to promote healthy eating and physical activity was increased after the *Romp and Chomp *intervention and although only a moderate level of capacity could be demonstrated by the end of the intervention phase, this was still regarded as substantial progress by those involved.

An essential aspect of capacity building is leadership [19,20], and although a clear project aim and specific objectives were agreed and articulated, we have found a strong perception of lack of leadership in the project on several levels. Leadership was consistently identified as an area of capacity building that was not addressed during the project and this finding leads us to recommend an investment in leadership training and strategies to increase group cohesion, team building, succession planning, collaboration and project management across the organisations involved. This is particularly important for the implementation of complex and long-term projects, such as *Romp & Chomp*, involving multiple partner and stakeholder organisations.

Challenges of limited resources and funding (AU$ 111,200 in total) across multiple agencies were major points of frustration for most of the key informants and it was thought to have directly affected project implementation. The implementation team appeared to have overcome the limited finances partly with resource reallocation and an increased degree of in-kind support and personal input and commitment. Previous research identifies these as important aspects of capacity building [2]. More transparent resource allocation and documented in-kind contributions may have reduced dissatisfaction and further enhanced collaboration between organisations.

A central intervention strategy in *Romp & Chomp *was professional development and workforce training (in nutrition and active play), which have been identified as important capacity building strategies in public health nutrition [21]. *Romp & Chomp *facilitated the training of future childcare workers in active play through the integration of professional development into the curriculum of the TAFE program. This was viewed as a sustainable and potentially cost-effective method of capacity building. The training of allied health professionals to support child care workers and early childhood settings staff to implement health promotion programs was also identified as a good outcome from the project. Although not captured in this evaluation, this aspect of the project has now become integrated into the larger, state-wide health promotion project (Kids-'Go for your life'), increasing its reach and sustainability. During the development of future health promotion projects the sustainability of the various capacity building activities should be planned to ensure ongoing benefits to the community after the specific health promotion project is completed.

There were distinct strategies in the project action plan to enhance organisational development; however, the findings from this study demonstrate that a number of important issues related to communication, roles and responsibilities, leadership and resources were not addressed. These issues were felt to have slowed down project implementation and strained relationships. This highlights the need to establish agreed structures and protocols early on in a complex project such as this, to ensure effective communication and clear roles and responsibilities across and within partner organisations. These structures and protocols should be reviewed periodically to ensure they are still appropriate for the stage of the project, given the often long term nature of these large scale projects. It is also important to assess the performance of the partnerships throughout the life of the project through a formal process and to address issues as they arise. Inter-organisational collaboration and partnerships are often complicated and can be difficult to manage and in addition to our experience here, previous research also suggests that strategies to foster strong collaborations and addressing the ongoing needs of partnerships should be a priority in these types of health promotion projects [5,22].

It is evident that a high performing, cohesive, clear and transparent partnership was not fully achieved through the implementation of the *Romp & Chomp *project. But despite the large number of barriers and challenges that were reported through this evaluation, our analysis of the qualitative data identified a genuine sense that a number of positive outcomes were achieved and that lasting attitudinal and policy changes have resulted across the Geelong community. Triangulation of the three data sources indicates that considerable improvements were made in the partnerships, knowledge transfer and problem solving domains, while the areas of capacity building related to policy, human/intellectual and social investments were not as well developed. The use of a capacity-building framework to determine the specific intervention strategies required may have avoided a number of the issues identified and highlighted the areas where there was little activity, providing an opportunity to address the gaps in the program.

### Limitations

There are a number of strengths and limitations of this evaluation study. One limitation was a lack of documentation for some aspects of the project implementation which means that we may have under-represented the activities in certain areas of the project. To overcome this problem however we used data triangulation rather than relying on only one method (which is a considerable strength of this study). Secondly there was no comparison group which could make it more difficult to determine if increased capacity was initiated by the project. However, this was overcome by the use of both qualitative and quantitative evaluation methods and in the interviews key informants were asked to reflect on changes over time that resulted from the intervention project specifically. Thirdly we have only assessed the impact of the project on community capacity directly after the end of the intervention and there may be additional 'spin offs' and flow-on effects from *Romp & Chomp *that were not identified in this evaluation or emerge in the future. Again this would result in an under-representation of the impacts of the intervention. Reassessment of community capacity in a few years will provide additional useful evidence of the sustainability of the increased community capacity in the Geelong community.

Based on our learnings we recommend the following:

• Intervention strategies and their evaluation should be guided by an appropriate theoretical framework such as a capacity building framework

• If taking a capacity building approach, ongoing activities are needed which address all aspects of capacity building with a focus on leadership skills within the implementation network

• Given the challenging nature of this approach, a commitment to long term efforts to foster and maintain collaborations and partnerships are required at all levels, from the individuals implementing the program to those high up in the stakeholder organisations involved

• Ongoing specific intervention activities are needed to foster and maintain the implementation network and partnerships

• Clarity around the roles and responsibilities of partner organisations and the recognition of their cash and in-kind contributions are important

• Ongoing evaluation of the performance of the network and partnerships is required

## Conclusions

*Romp & Chomp *increased the capacity of organisations, settings and services in the Geelong community to support healthy eating and physical activity for young children. Despite this success there are important learnings related to project management, leadership, governance, communication, documentation, capacity, resources, collaboration and fostering strong partnerships that should be addressed in future long-term, community-based health promotion projects of this kind. Adopting these recommendations should strengthen the capacity of stakeholder organisations to implement efforts to improve children's health and support families in their endeavours also. As one of the interviewees stated:

*"Despite the difficulties...that kind of collaboration is what we need to do more and we just need to get better at it"*.

## Competing interests

The authors declare that they have no competing interests.

## Authors' contributions

FPG participated in the data collection and carried out the statistical analyses and interpretation, and drafted the manuscript. NMR participated in the data collection and revised the manuscript. BS was involved in designing the study and revising the manuscript. AdS was involved in designing the study, the interpretation of the data and drafting the manuscript. All authors read and approved the final manuscript.

## Pre-publication history

The pre-publication history for this paper can be accessed here:

http://www.biomedcentral.com/1471-2458/10/522/prepub
